# Using a bioluminescence resonance energy transfer caspase biosensor to study caspase-3 cleavage site specificity

**DOI:** 10.1042/BSR20254030

**Published:** 2026-03-24

**Authors:** Véronique Blais, Jean-Bernard Denault

**Affiliations:** 1 Department of Pharmacology and Physiology, Faculty of Medicine and Health Sciences, Université de Sherbrooke, Sherbrooke, Québec J1H 5N4, Canada; 2 Institut de Pharmacologie de Sherbrooke, Université de Sherbrooke, Sherbrooke, Québec J1H 5N4, Canada; 3 Centre de recherche du CHUS, Sherbrooke, Québec J1H 5N4, Canada

**Keywords:** bioluminescence resonance energy transfer (BRET), biosensor, peptidases

## Abstract

In its simplest representation, apoptosis is a two-step peptidase cascade in which initiator caspases (caspases-8, -9, and -10) activate executioner caspases (caspases-3, -6, and -7). Although many intricacies exist—such as the proteolysis of initiator caspases by executioner caspases, which further regulates their activity—apoptotic pathways ultimately converge on the activation of caspase-3, the most proteolytically proficient member of the family. This central role has led to the development of numerous enzymatic assays to detect caspase-3 activity, its activation, and the cleavage of hallmark substrates, such as poly(ADP-ribose) polymerase 1. Like other members of the caspase family, caspase-3 minimally recognizes a five-amino-acid motif, usually located in a well-exposed loop within its substrates. Caspase-3 cleavage-site motif preferences have been systematically studied using peptides but not proteins. Here, we use a simple recombinant protein-based double brilliance bioluminescence resonance energy transfer (BRET^2^) biosensor assay for caspase-3 that enables robust and quantitative kinetic measurements *in vitro*. We used the biosensor by assessing its ability to distinguish between optimal and suboptimal cleavage-site motifs using a panel of BRET^2^ biosensors incorporating all 20 amino acids at the critical P_4_ position (the fourth residue N-terminal to the scissile bond). Except for arginine and lysine, we successfully determined the catalytic specificity (*k*_cat_/*K*_M_) for all other residues at P_4_. Notably, the range of proteolytic efficacies observed with BRET biosensors was significantly narrower than that previously reported using peptide-based libraries. Finally, we confirmed the biosensor’s utility in apoptotic cells, demonstrating its robustness and broad applicability.

## Introduction

In humans, caspases are a family of 11 active cysteinyl peptidases involved in apoptosis, cytokine maturation, epithelial differentiation, and several non-apoptotic processes, including neuronal plasticity, stem cell differentiation, and immune regulation [1–4]. During apoptosis, a two-step peptidase cascade occurs in which initiator caspases (caspases-8, -9, and -10) activate executioner caspases (caspases-3, -6, and -7) [5]. The first step involves the activation of at least one initiator caspase via either the intrinsic or extrinsic pathway. The intrinsic pathway, triggered by internal stress signals, involves mitochondrial release of cytochrome *c*, formation of the apoptosome [6–10], and activation of caspase-9 by dimerization [11–17]. Conversely, the extrinsic pathway relies on death receptor ligation by immune cell-derived agonists [18–20], leading to assembly of the death-inducing signaling complex and dimerization and activation of caspases-8 and -10 [17,21–26]. In a common second step, active initiator caspases cleave pre-dimerized executioner caspases-3 and -7 [27,28], and caspase-3 subsequently activates caspase-6 by proteolysis [27,28]. Executioner caspases, particularly caspase-3, drive the characteristic biochemical changes of apoptosis, including cleavage of poly(ADP-ribose) polymerase 1 (PARP1), exposure of phosphatidylserine, and fragmentation of genomic DNA—events that ultimately lead to cell death [29–31]. Although many cell-specific intricacies exist, the activation of caspase-3 and the cleavage of *bona fide* substrates, like PARP1, remain hallmarks of apoptosis and are often used as a readout [32–35].

Caspases have distinct, yet overlapping, preferences for the motifs they recognize within their substrates. Biochemical characterization of their preferences has primarily used short peptidic substrates in combinatorial libraries containing natural amino acids [36–42] as well as unnatural ones [43]. Other approaches have employed proteomics to study caspase preferences by surveying the caspase degradome, i.e., repertoire of protease substrates [44–47]. Collectively, these studies have shown that caspase-3 and its close homolog caspase-7 prefer the DEVD↓(G/A/S) motif (P_4_-P_3_-P_2_-P_1_↓P_1′_ Schechter and Berger cleavage site nomenclature [48]). Aside from the universally preferred P_1_-Asp and P_1′_-Gly/Ala/Ser residues, the P_4_ amino acid is key in distinguishing cleavage-site preference among caspases. In addition, determinants beyond these five residues may contribute to efficacious proteolysis, such as the extended caspase-2 substrate-binding site accommodating a P_5_-Val [36], as well as more distant features like exosites (reviewed in [49]). Despite different preferences among caspases, caspase-3 remains the most active family member on most, if not all, peptidic motifs preferred by other caspases [50].

Many genuine caspase substrates required for complete and rapid apoptosis bear optimal or near-optimal motifs. For example, PARP1 is cleaved at an optimal DEVD↓G motif by caspases-3 and -7, and caspase-3 is activated by initiator caspase-8 at an optimal IETD↓S sequence. Nevertheless, notable exceptions exist in which suboptimal cleavage sites are used, such as the proteolysis of another apoptotic hallmark HSP90 co-chaperone p23 by caspase-7 [51], which is cleaved at a PEVD↓G motif (P_4_-Pro).

Knowledge of the primary structure recognized by caspases has led to the development of sensitive substrates and inhibitors. Commercially available caspase detection kits typically comprise a small peptidic substrate incorporating an optimal caspase-3/7 motif that releases a colored, fluorescent, or luciferase substrate moiety upon proteolysis. More bespoke molecules employ library-compatible fluorophores, such as 7-amino-4-carbamoylmethylcoumarin (ACC), and unnatural amino acids to improve sensitivity and selectivity among caspases [43]. Another type of peptide-based substrate consists of internally quenched molecules presenting residues on both sides of the scissile bond [52,53]. In intact cells, caspase detection methods include the use of cell-permeable, fluorescent, mechanism-based irreversible inhibitors that label active caspases (e.g., fluorochrome-labeled inhibitors of caspases/FLICA [54]).

Most, if not all, small peptide-based assays are light-on substrates, but light-off caspase reporters also exist, including Förster resonance energy transfer (FRET) molecules. Several FRET sensors have been developed, many of them using fluorescent proteins that are particularly useful for live-cell imaging because they can be genetically encoded [55,56]. A FRET biosensor detects proteolysis by measuring the loss of energy transfer between a donor and acceptor fluorophore since this distance-dependent transfer sharply decreases upon cleavage [57–59]. Such FRET-based biosensors bearing caspase cleavage sites have been successfully used to model apoptosis in intact cells [60–62]. Direct light-on biosensors have also been developed in recent years, like iCasper [63], engineered cyclic mNeonGreen2-DnaE intein [64], and split-luciferase reporters [65].

Bioluminescence resonance energy transfer (BRET) has become increasingly popular for studying biological processes, including proteolytic activities [66–68], because it offers lower background noise and avoids photobleaching compared with FRET-based biosensors [69]. Notable adaptations for caspase activity detection include a NanoLuc–mNeonGreen pair for monitoring caspases-3, -8, and -9 *in vitro* and in cells [68] and a red-shifted BRET pair combining click beetle green luciferase with tdTomato for real-time tracking of caspase-3 activity in living cells [70].

Very few studies have determined the kinetics of cleavage efficacy (*k*_cat_/*K*_M_) of caspases using proteinaceous substrates [36,38,47], and none has done so systematically using a protein substrate that has not co-evolved with caspases, i.e., an unbiased substrate. Here, we use a simple and highly adaptable double-brilliance recombinant protein BRET^2^-based GFP10–*R*LucII caspase biosensor to study caspase-3 substrate preference. This biosensor enables the measurement of the catalytic specificity of proteolysis in a single assay under pseudo-first-order conditions, allowing robust and reproducible comparisons of cleavage sites. It also enables caspase activity detection when the biosensor is expressed in cells.

## Materials and methods

### Chemicals and reagents

Coelenterazine 400a (cat: C-320) was from Gold Biotechnology Inc.; etoposide (VP-16; cat: 341205), imidazole (cat: I-0250), and dithiothreitol (DTT; cat: D-0632) were from Millipore Sigma; iodoacetamide (IAA; cat: AC122270050) was from Thermo Fisher Scientific; isopropyl β-d-1-thiogalactopyranoside (IPTG; cat: IB0168) was from Bio Basic Inc.; trypsin/Lys-C Mix (cat: V5071) was from Promega; the pan-caspase inhibitor Z-VAD-fluoromethyl ketone (Z-VAD-fmk; cat: ALX-260-020), caspases-3 and -7 fluorogenic substrate acetyl-DEVD-4-amino-7-fluorocoumarine (AcDEVD-Afc; cat: ALX-260-032) for caspase titration, and recombinant human soluble killer TRAIL (cat: ALX-201-073-3020) were from Enzo Life Sciences; M_r_ 25,000 linear polyethyleneimine (PEI; cat: 23966) was from Polysciences; and staurosporine (STS; cat: 81590-500) was from Cayman Chemical Co. All cell culture reagents were from Wisent Inc. General chemicals were from Millipore Sigma, Thermo Fisher Scientific, or Wisent Inc.

### DNA constructs

The BRET^2^-based GFP10–linker–*R*LucII biosensor [71] construct comprises residues 3–239 from GFP (UniProt: P42212; F303S/L232H substitutions) and amino acids 1–313 from *R*LucII (UniProt: P27652; T55A substitution) separated by a 17-residue linker (GSAGTGGRAIDIKLPAT). Caspase cleavage sites were generated by inserting small double-stranded DNA fragments into the KpnI and HindIII restriction sites of plasmid pET-23b(+) or pcDNA3.1(+) in which the BRET^2^ biosensor had been previously inserted using NdeI/XhoI or NheI/EcoRI, respectively. The short DNA fragments were generated by annealing 10 μM of the following phosphorylated oligonucleotides in 50 mM NaCl: 5′-c(Xxx)gaagtggatggcggca-3′ (forward) and 5′-agcttgccgccatcgagttc(Xxx)ggtac-3′ (reverse), in which Xxx represents a codon (or reverse complement) for each individual amino acid in P_4_ (Ala, gcc; Cys, tgc; Asp, gac; Glu, gaa; Phe, ttc; Gly, ggc; His, cac; Ile, atc; Lys, aag; Leu, ctg; Met, atg; Asn, aac; Pro, ccg; Gln, cag; Arg, cgc; Ser, agc; Thr, acc; Val, gtg; Trp, tgg; or Tyr, tac). The control biosensor DEVA was generated using a similar approach and oligonucleotides 5′-cgacgaagtggccggcggca-3′ (forward) and 5′-agcttgccgcccgccacttcgtcggtac-3′ (reverse).

Caspases-3 and -7 expression plasmids encoding a C-terminal His-tag were described elsewhere [72].

### Protein expression

Proteins were expressed as C-terminal 6His-tagged fusion proteins in BL21(DE3) p*LysS Escherichia coli* as described elsewhere [72]. Bacteria were grown in 2× YT medium with 50 μg ml^−1^ ampicillin to Abs_600_ 0.5 at 37°C, after which protein expression was induced with 0.2 mM IPTG at 30°C for 20 h. Bacteria were harvested by centrifugation and lysed by sonication in resuspension buffer (50 nM Tris, pH 8.0, and 100 mM NaCl). After clarification by centrifugation and filtration through a low protein-binding 0.45 μm membrane, bacterial lysates were applied to an immobilized metal affinity chromatography (IMAC) column (0.5–1.0 ml resin; Cytiva Life Science), washed with 10 column volumes of resuspension buffer, followed by 50 column volumes of washing buffer (50 nM Tris, pH 8.0, and 500 mM NaCl). Proteins were eluted with a 0–200 mM imidazole gradient using an AKTA prime fast protein liquid chromatograph (Cytiva Life Science).

Caspases-3 and -7 were active site-titrated using the mechanism-based inhibitor Z-VAD-fmk and Ac-DEVD-AFC fluorogenic substrate as previously described [73] to determine the active site concentration. Biosensor titration was achieved using initial absorbance at 280 nm and its extinction coefficient (ε_280_ = ∼85,510 M^−1^ cm^−1^), then GFP10 fluorescence (EX_λ_, 395 nm; EM_λ_, 510 nm) was compared with a purified GFP10 reference preparation. Biosensor purity was estimated using a GelCode Blue-stained SDS–PAGE image and Image Lab v6.1 software (Bio-Rad). The purity of preparations was assessed using the total protein normalization method, following the manufacturer’s instructions.

### *In vitro* BRET^2^ assays

Unless specified otherwise in the figure legends, BRET^2^ assays were performed in a white 96-well plate using 10 nM biosensor, 25–500 nM active site-titrated pre-activated (20 min with 10 mM DTT at 30°C) caspase-3 or caspase-7, and 5 μM coelenterazine 400a luciferase substrate in assay buffer (10 mM piperazine-*N*,*N′*-bis(2-hydroxypropanesulfonic acid)/PIPES, pH 7.2, 100 mM NaCl, 10% sucrose, 0.1% (w/v) 3-((3-cholamidopropyl)-dimethylammonio)-1-propanesulfonate/CHAPS, 1 mM EDTA, and 10 mM DTT) at 37°C.

The BRET^2^ signal was measured over 30 min using a TECAN M1000 plate reader. *R*LucII and GFP10 emissions were collected in the 400–450- and 500–550-nm windows, respectively. The BRET^2^ ratio was calculated as the intensity of the light emitted by the acceptor GFP10 over the light emitted by the donor *R*LucII. All data were analyzed using nonlinear curve fitting in GraphPad Prism software v10.5.0 (see below). Measurements were performed in 3–4 technical replicates. For kinetic measurements (P_4_ library), results are expressed as means ± SEM from independent replicates. The numbers of independent experiments *N* are indicated in the figure legends.

### Kinetic analysis

**Figure 1:**
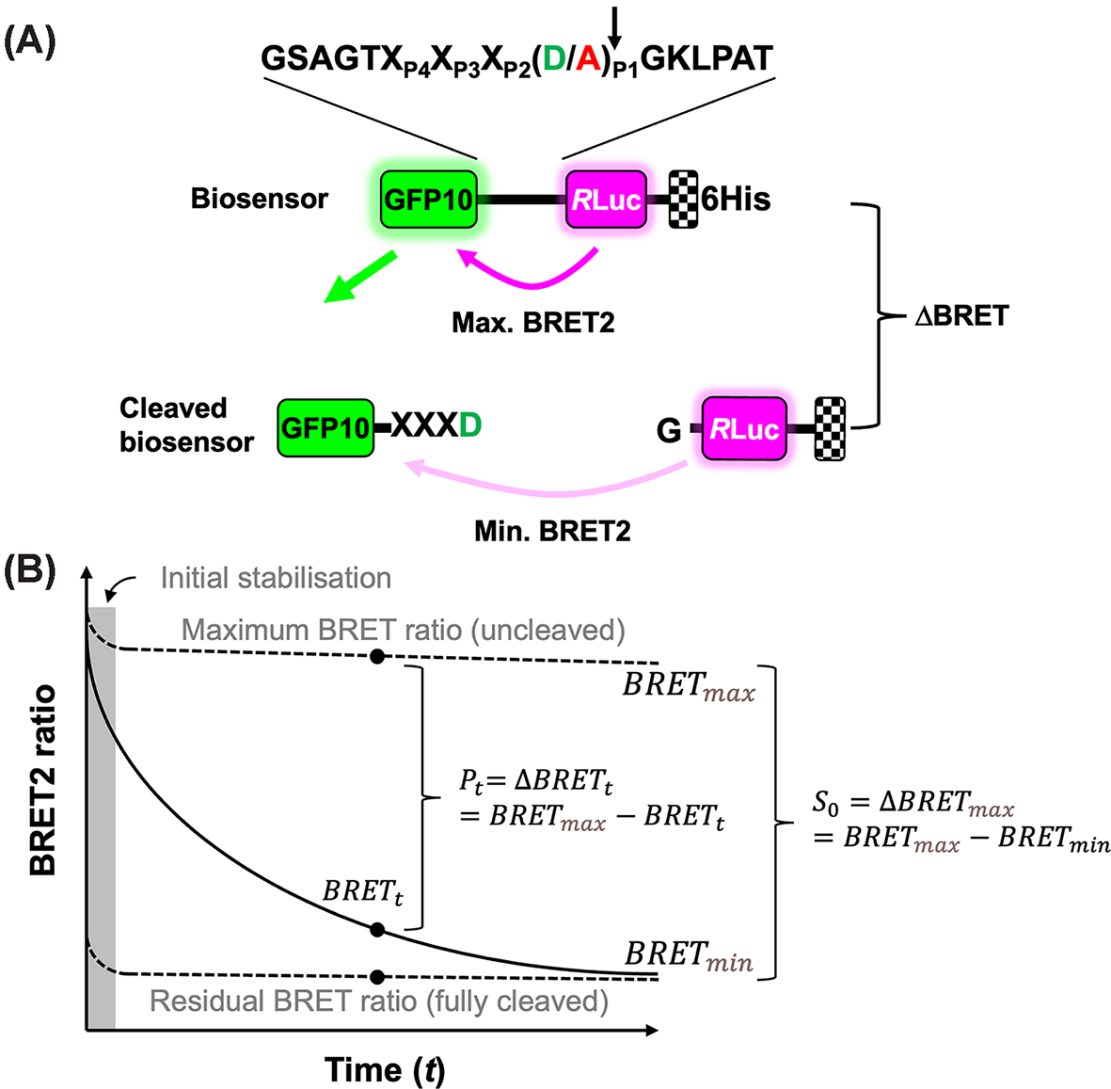
BRET^2^ caspase biosensor design (**A)** Schematic representation of the BRET^2^ caspase biosensor with a generic caspase cleavage site (arrow) separating GFP10 and *R*LucII proteins. Efficient energy transfer occurs when GFP10 is within 100 Å but is lost following proteolysis within the linker. (**B**) Theoretical trace of the decrease in BRET^2^ ratio caused by the cleavage of the biosensor. Over time (*t*), the BRET^2^ signal ratio observed (continuous trace) is comprised between a maximal ratio (uncleaved; *BRET*_max_) and a minimal ratio (fully cleaved; BRET_min_) proportional to total initial substrate (S_0_) concentration or the maximal BRET^2^ ratio change (∆BRET_max_). See the ‘Material and methods’ section for details.

The engineered BRET^2^ biosensor incorporates caspase cleavage sites between the GFP10 and the *R*LucII luciferase protein (Figure 1A), resulting in maximal signal when intact and minimal signal once cleaved. To quantify biosensor cleavage and obtain cleavage rates, we used the pseudo-first-order kinetic equation:

(1) $$P_{t}=S_{0}\left(1-e^{-kE_{0}t}\right)$$

describing the accumulation of product over time (*P_t_*) from an initial substrate concentration (*S_0_*) as a function of time (*t*), enzyme concentration (*E_0_*), and a rate constant *k* approximating the second-order rate constant *k*_cat_/*K*_M_. This rate equation is valid if substrate concentration is much below *K_M_*, which is a reasonable assumption at a biosensor concentration of 10 nM. In the BRET^2^ assay (Figure 1B), the total substrate signal (i.e*.*, max BRET^2^ signal variation, *S_0_*) is given by

(2) $$S_{0}=∆{\mathrm{BRET}}_{\max}={\mathrm{BRET}}_{\max}-{\mathrm{BRET}}_{\min},$$

which is constant during assay time, unless the biosensor is cleaved or degraded, GFP10 bleaches (unlikely in BRET), or the *R*LucII activity is lost. Conversely, the cleaved biosensor signal at time *t* (product BRET^2^ signal, *P_t_*) is

(3) $$P_{t}=∆{\mathrm{BRET}}_{t}={\mathrm{BRET}}_{\max}-{\mathrm{BRET}}_{t}.$$

Replacing *P_t_* and *S_0_* in (eqn 1) yields:

(4) $$\left({\mathrm{BRET}}_{\max}-{\mathrm{BRET}}_{t}\right)=\left({\mathrm{BRET}}_{\max}-{\mathrm{BRET}}_{\min}\right)\left(1-e^{-kE_{0}t}\right).$$

Isolating *BRET_t_*, which is what is being measured, gives:

(5) $${\mathrm{BRET}}_{t}=\left({\mathrm{BRET}}_{\min}-{\mathrm{BRET}}_{\max}\right)\left(1-e^{-kE_{0}t}\right)+{\mathrm{BRET}}_{\max}.$$

For the time course of BRET^2^ ratio analysis, initial values for BRET_max_ and BRET_min_ were estimated as the maximal signal observed during the assay (usually the signal at *t* = 0) and 5% of that maximal signal, respectively, and *k* to a value of 1000 M^−1^ s^−1^, BRET_max_ and *k* were constrained to greater than zero. BRET_min_ was allowed to go negative to help fit the data from poor substrates (incompletely hydrolyzed with no observed data flattening) and prevent overestimation of *k* values. BRET^2^ values are collected following a short lag (<90 s) during which the assay components are assembled and mixed, and the signal stabilizes (shaded area, Figure 1B). Because the analysis deals with the change in the rate of product generation over time, which is characterized by *k*, it is insensitive to the initial substrate concentration if pseudo-first-order conditions are met. For this reason, hydrolysis of the biosensor prior to the first read does not affect the analysis. For presentation purposes, each dataset was normalized to BRET_max_, which was obtained by an initial analysis; normalization does not affect *k* values.

### Statistical analyses

Statistical analyses were performed using GraphPad Prism software. Data normality was assessed using the Shapiro–Wilk test. Since normally distributed data was confirmed, a one-way ANOVA was applied, followed by Dunnett’s multiple comparison test to compare groups with the control group. Results were considered statistically significant at *P* <0.05 (*), *P* <0.01 (**), *P* <0.001 (***), or *P* <0.0001 (****).

### Mass spectrometry analysis

Samples were thawed on ice prior to processing. Proteins were denatured by adding 6 M urea in 25 mM Tris, pH 8.0, reduced by adding 10 mM DTT in 25 mM Tris, pH 8.0, and incubated at 65°C for 10 min. Alkylation was carried out by adding 15 mM IAA in 25 mM Tris, pH 8.0, and incubation for 30 min at room temperature in the dark. The alkylation reaction was quenched by adding an additional 10 mM DTT. Proteins were precipitated using 5 volumes of cold acetone, followed by incubation at −80°C for 2 h. Then, samples were centrifuged at 10,000×***g*** for 10 min at 4°C, and the supernatant was discarded. Protein precipitates were vacuum-dried for 5 min to remove residual acetone and resuspended in 50 μl of 50 mM Tris, pH 8.0. Proteins were digested with 1 μg of trypsin/Lys-C Mix at 37°C for 2 h. An additional 1 μg of trypsin/Lys-C was added, and digestion continued for 16 h at 37°C. Digestion was stopped by acidifying the samples with formic acid to a final concentration of 2% (v/v).

The loading and elution steps were done by gravity. Samples were pre-treated with 150 μl of 4% H_3_PO_4_, then loaded onto an HLB Prime multi-well plate (Waters). Wells were washed with 5% methanol, dried using a nitrogen stream, and proteins were eluted four times with 50 μl of a MeCN:MeOH:TFA mixture (90:10:1). Eluates were dried under vacuum in a low-binding polypropylene tube and reconstituted in 50 μl of water:MeCN:formic acid mixture (98:2:0.1). Samples were centrifuged at 10,000×***g*** for 5 min at 4°C and transferred into low-binding LC-MS vials. Peptides were analyzed on a QTRAP 6500+ mass spectrometer (SCIEX) coupled to a BEH C18 peptide column (100 × 1 mm, 1.7 μm). The column temperature was maintained at 40°C with a flow rate of 100 μl min^−1^, and the injection volume was 3 μl. The mobile phase consisted of 0.1% formic acid in water (A) and 0.1% formic acid in MeCN (B). Mass spectrometric detection was performed in positive electrospray ionization mode (ESI^+^) using multiple reaction monitoring (MRM). *In silico* MRM transitions were generated in Skyline (MacCoss Lab Software) with doubly (+2) and triply charged (+3) precursors selected and corresponding y- and b- type fragments ions monitored. Data acquisition was carried out using Analyst software v1.4.6 (SCIEX).

### Cell culture and transfection

Fetal embryonic kidney AD-293 (240085; Agilent Technologies) and human umbilical vein EA.hy926 cells (ATCC; CRL-2922) were cultured in DMEM medium supplemented with 10% fetal bovine serum (FBS), 2 mM l-glutamine, 100 IU ml^−1^ penicillin, and 100 μg ml^−1^ streptomycin. For transfection, 2 × 10^5^ cells were transfected with 3 μg biosensor plasmid using 9 μg PEI (prepared as 1 mg ml^−1^ in distilled water) in 150 mM NaCl. Twenty-four hour post-transfection, media were replaced with media without FBS for 1 h, and cells were left untreated or exposed to TRAIL, STS, or VP-16 at the indicated concentration and for the indicated period or were irradiated with UV light with the indicated dose and time.

### Protein analysis

Cells were harvested by mechanical resuspension using PBS and centrifuged. Protein lysates were prepared in mRIPA buffer (50 mM Tris, pH 8.0, 100 mM NaCl, 1% (v/v) NP-40, 0.5% deoxycholic acid, 0.1% SDS, and 1 mM EDTA) and quantified using the Pierce BCA protein assay kit (Thermo Fisher Scientific). Samples were separated on 10% tris-glycine polyacrylamide gels (SDS–PAGE) and stained with GelCode Blue (Thermo Fisher Scientific).

Alternatively, proteins were separated by SDS–PAGE and analyzed by immunoblotting on a PVDF membrane (Millipore Sigma) using CAPS/MeOH (10 mM CAPS, pH 10; 10% MeOH) buffer for transfer [74]. PVDF membranes were sequentially blocked with 5% non-fat dry milk (Carnation) in PBS plus 0.1% Tween, anti-GFP (A11122; Thermo Fisher Scientific, dilution:1:3000), anti-PARP1 (9532S; New England Biolabs, dilution: 1:7500), or anti-actin (A3853; Millipore Sigma, dilution: 1:5000) primary antibody in 3% BSA in PBS, and horseradish peroxidase (HRP)-conjugated secondary antibody in 5% milk PBS–Tween (anti-mouse (7076S), anti-rabbit (7074S); New England Biolabs, dilution: 1:5000). HRP was revealed using Immobilon Crescendo Western HRP Substrate chemiluminescence reagent (Millipore Sigma). Non-saturated images were acquired using a VersaDoc 4000 MP or ChemiDoc imaging system (Bio-Rad).

## Results

### BRET^2^ caspase biosensor design

To generate a BRET^2^ biosensor capable of reporting caspase activity, we engineered a bacterially expressed GFP10–*R*LucII fusion protein separated by a linker that can be replaced with a segment containing caspase cleavage sites, yielding a 15-residue linker (Figure 1A). When intact and uncleaved, a maximal BRET^2^ ratio is observed due to the proximity of GFP10 and *R*LucII, whereas a minimal BRET^2^ ratio is measured following proteolytic separation. The kinetics of BRET^2^ ratio decrease can be modeled using an equation incorporating pseudo-first-order kinetics, $P_{t}=S_{0}(1-e^{-kE_{0}t})$ in which the second-order rate constant *k* estimates *k*_cat_/*K*_M_ when the substrate concentration is much lower than *K_M_* (Figure 1B; see the ‘Materials and methods’ section). Thus, once experimental conditions are optimized, this assay provides a measurement of catalytic specificity in a single experiment and allows quantitative discrimination between cleavage sites in a proteinaceous substrate that has not co-evolved with the peptidase.

### Assessing the BRET^2^ caspase biosensor

**Figure 2:**
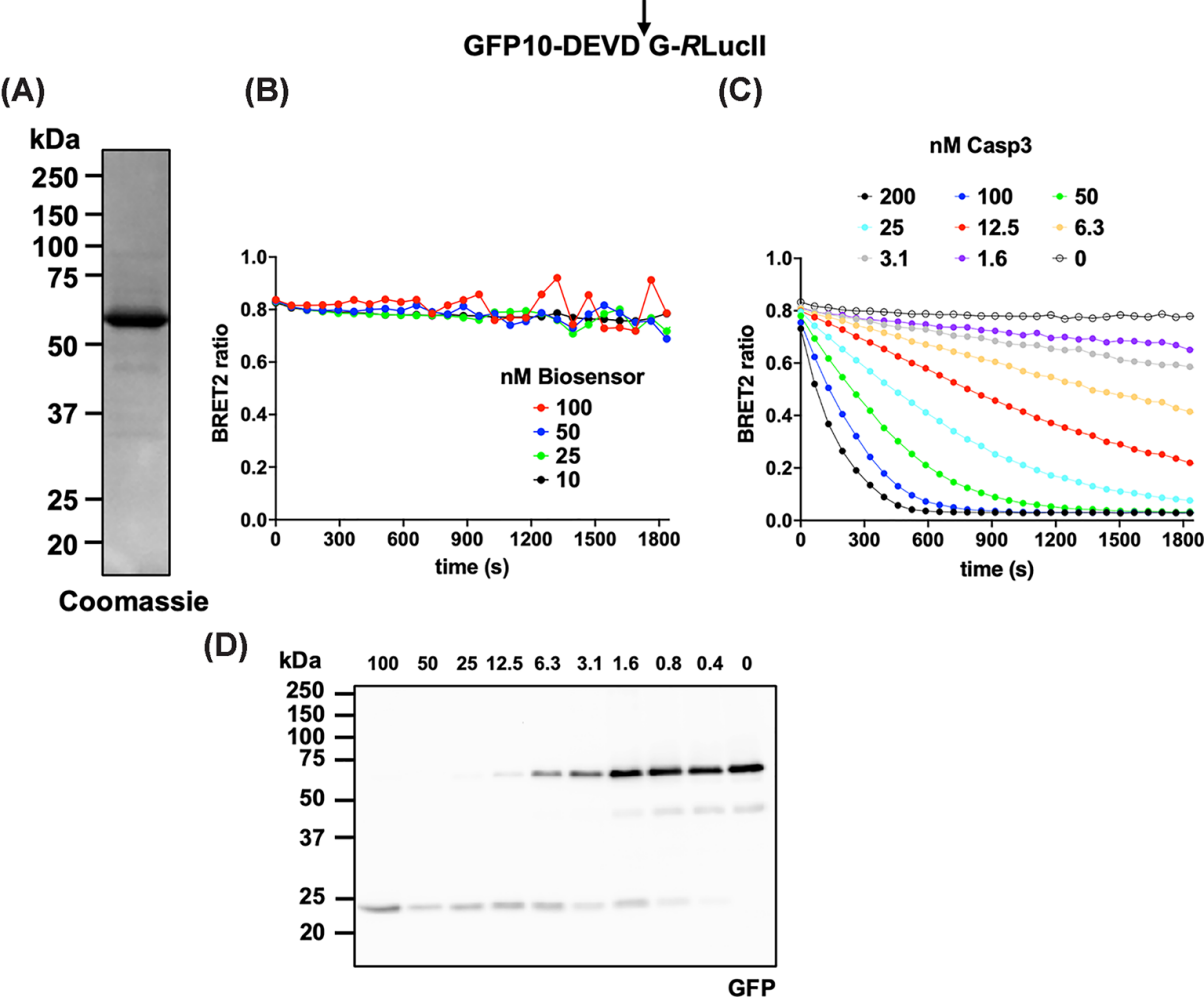
DEVD biosensor (**A**) Expression and purification of the BRET^2^ DEVD biosensor. Five μg of IMAC-purified biosensor was analyzed by SDS–PAGE. (**B**) BRET^2^ signal of intact DEVD biosensor over 30 min obtained with different concentrations of the biosensor. (**C**) Caspase-3 concentration-dependent reduction in BRET^2^ signal over time. Ten nM of DEVD biosensors were incubated with the indicated caspase-3 concentration, and the BRET^2^ signal was monitored over 30 min. (**D**) Caspase-3 concentration-dependent cleavage of the DEVD biosensor. Fifty nM of DEVD biosensors were incubated with the indicated caspase-3 concentration for 60 min. Samples were analyzed by immunoblotting with anti-GFP antibody. Results are representative of four (A), three (B), and two (C, D) independent experiments.

The BRET^2^ biosensor bearing the caspase-3-preferred DEVD↓G motif (DEVD biosensor) was expressed in *E. coli* and purified using IMAC to ∼83% protein purity (Figure 2A and Supplementary Figure S1). This purity level is adequate, as similar kinetic results were obtained using unpurified biosensors from lysates (Supplementary Figure S2), demonstrating the robustness of the assay.

For the BRET^2^ biosensor assay to function optimally, three important parameters were optimized: biosensor concentration, caspase concentration, and assay duration. In samples lacking peptidase, the BRET^2^ signal remained stable for at least 30 min using 10–50 nM biosensor and 5 μM coelenterazine 400a, the *R*LucII substrate, but became unstable at biosensor concentrations of 100 nM and above (Figure 2B). A 30-min acquisition period was sufficient to generate more than 30 data points for kinetic analysis. To satisfy pseudo-first-order kinetic conditions, we selected a biosensor concentration of 10 nM, which is well below the estimated *K_M_* value, yet provides a strong BRET^2^ signal that can be reliably quantified—an important consideration given that this is a light-off sensor (see the ‘Discussion’ section). *K_M_* values for most peptidases exceed 1 μM [75], and for rapidly cleaved caspase substrates such as PARP1, *k*_cat_/*K*_M_ is approximately 1 × 10^7^ M^−1^ s^−1^ [76]. Thus, *K_M_* is expected to remain greater than 100 nM when *k_cat_* exceeds 1 s^−1^ [76]. We assessed the caspase-3 concentration required to cleave the DEVD biosensor (Figure 2C). Varying caspase-3 concentrations from 1.6 to 200 nM resulted in a concentration-dependent decrease in the BRET^2^ signal, with complete loss of the BRET^2^ ratio within 12 min at 100 nM and partial but detectable reduction at 1.6 nM. Parallel assays analyzed by SDS–PAGE (Figure 2D) showed approximately 50% biosensor cleavage after 1 h using 3.1 nM caspase-3, which is consistent with the ∼25% BRET^2^ signal loss observed within 30 min for the same caspase concentration.

**Figure 3:**
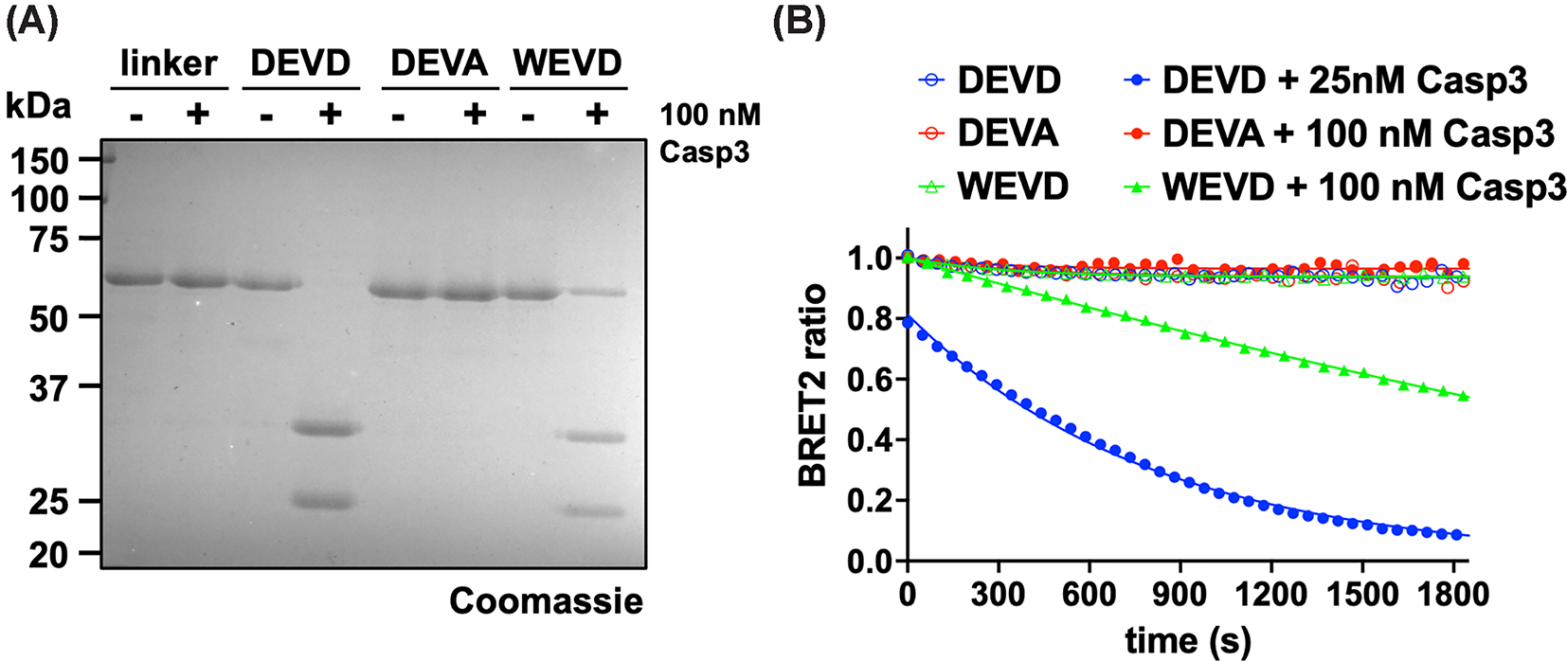
Caspase-3 optimized biosensor (**A**) One μg of purified biosensor presenting an irrelevant linker (GSAGTGGRAIDIKLPAT), DEVD (caspase-3-optimized site), DEVA (uncleavable motif), or WEVD (sub-optimal motif) was incubated without or with 100 nM caspase-3 for 75 min and analyzed by SDS–PAGE. (**B**) BRET^2^ signal of indicated biosensors (10 nM) incubated with 25 nM (DEVD) or 100 nM (DEVA and WEVD) caspase-3 for 30 min. Results are representative of three (A) and two (B) independent experiments.

To assess the range of substrate motifs that can be analyzed using the BRET^2^ biosensor, we compared the preferred caspase-3 recognition motif, DEVD↓G, with WEVD↓G (WEVD), which features a P_4_-Trp residue optimal for inflammatory caspases (Figure 3) [42]. Incubation of 1 μg (750 nM) of biosensor containing the DEVD, WEHD, or no caspase motif with 100 nM caspase-3 for 75 min revealed efficacious cleavage of the DEVD-containing biosensor, minimal cleavage of the WEHD-containing biosensor, and no cleavage of the control biosensor (Figure 3A). A biosensor containing a non-cleavable DEVA motif (P_1_-Ala) was also included as a control, confirming that cleavage was caspase-dependent and occurred at the P_1_–P_1′_ peptide bond.

In kinetic assays, we observed similar results: the DEVD-containing biosensor was rapidly cleaved (with a fraction of the biosensor already cleaved before the first measurement), whereas the WEHD-containing biosensor was cleaved more slowly, necessitating an increase in caspase-3 concentration to achieve partial cleavage over the same period (Figure 3B). Analysis of similar data using a pseudo-first-order kinetic equation for the loss of the BRET^2^ ratio signal (see the ‘Materials and methods’ section) revealed a 35-fold higher cleavage efficacy for the DEVD-containing biosensor than for the WEVD-containing biosensor (DEVD: *k*_cat_/*K*_M_ = 59,000 ± 1400 M^−1^ s^−1^, WEHD: *k*_cat_/*K*_M_ = 1700 ± 100 M^−1^ s^−1^, see next section).

### *In vitro* use of caspase biosensors

**Figure 4:**
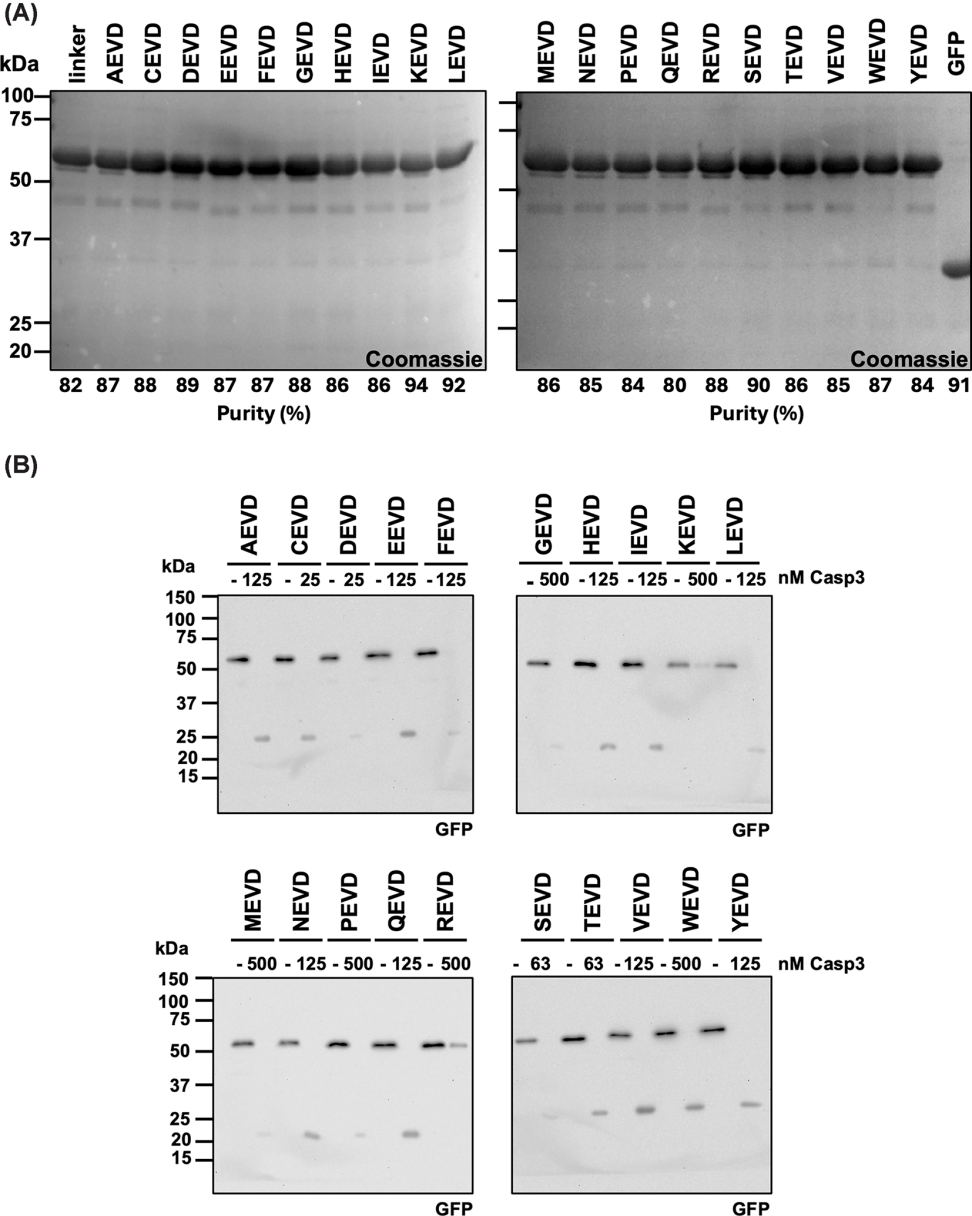
P_4_ caspase biosensor library (**A**) Five μg of each purified (P_4_X)EVD biosensor and GFP were analyzed by SDS–PAGE. (**B**) Thirty ng (10 nM) of each (P_4_X)EVD biosensor were incubated for 30 min with the indicated optimized concentration of caspase-3, resulting in full cleavage of the biosensor—except for the KEVD and REVD biosensors—and analyzed by immunoblotting using an anti-GFP antibody. (**C**) Calculated *k* values for the cleavage by caspase-3 of each (P_4_X)EVD biosensor using assays as described in Figure 3B. A full set of BRET^2^ ratio graphs is shown in Supplementary Figure S3 and compiled in Table 1. *n* = 6. Results are representative of four (A) and two (B) independent experiments.

Earlier work on caspase cleavage site preference has shown that caspase-3 strongly prefers a P_4_-Asp [41,42] but can accommodate other residues sufficiently well that this caspase outperforms others even on their respective preferred motifs [50]. To demonstrate the ability of the BRET^2^ biosensor to quantitatively differentiate the contribution of the P_4_ residue to cleavage efficacy, we generated a series of biosensors containing each of the 20 amino acids at this position (Figure 4A). The average purity of all P_4_ biosensors was estimated at 87 ± 3% (range 80%–94%).

This series of biosensors was incubated with an optimized concentration of caspase-3 that achieved complete cleavage within 30 min and analyzed by SDS–PAGE to assess cleavability (Figure 4B). Except for the KEVD and REVD biosensors, all biosensors were fully cleaved, indicating that none of them were purified as aggregates or misfolded proteins with an inaccessible cleavage site. Qualitatively, the results show that the biosensors segregate into five groups based on proteolytic efficacy, defined by the concentration of caspase-3 required to achieve complete cleavage: (1) CEVD and DEVD biosensors, 25 nM caspase-3; (2) SEVD and TEVD biosensors, 63 nM caspase-3; (3) AEVD, EEVD, FEVD, HEVD, IEVD, LEVD, NEVD, QEVD, VEVD, and YEVD biosensors, 125 nM caspase-3; (4) GEVD, MEVD, PEVD, and WEVD biosensors, 500 nM caspase-3; and (5) KEVD and REVD biosensors, which were not fully cleaved at 500 nM caspase-3.

In kinetics assays (Figure 4C, Supplementary Figure S3, and Table 1), the P_4_-Asp-containing biosensor was the most efficaciously cleaved, with a *k*_cat_/*K*_M_ value of 59,000 ± 1400 M^−1^ s^−1^. The P_4_-Cys-containing biosensor ranked second in cleavage efficacy, with a *k*_cat_/*K*_M_ value of 34,400 ± 1000 M^−1^ s^−1^ (58% that of the P_4_-Asp biosensor).

**Table 1:** Cleavage efficacy of the P_4_ series of BRET^2^ biosensors by caspase-3

Motifs	*K*_cat_/*K*_M_ (M^−1^ s^−1^) ± SEM	Fold less than DEVD^*^
DEVD	59,000 ± 1000	1.0
CEVD	34,000 ± 1000	1.7
TEVD	28,000 ± 800	2.1
SEVD	26,000 ± 500	2.3
HEVD	18,000 ± 500	3.3
EEVD	17,000 ± 300	3.5
VEVD	17,000 ± 300	3.5
NEVD	16,000 ± 700	3.7
AEVD	13,000 ± 400	4.5
IEVD	11,000 ± 200	5.4
YEVD	8800 ± 100	6.7
QEVD	7800 ± 200	7.6
FEVD	6400 ± 100	9.2
LEVD	6000 ± 100	9.8
PEVD	4200 ± 200	14.0
MEVD	2300 ± 100	25.7
GEVD	2200 ± 100	26.9
WEVD	1700 ± 100	34.7
KEVD	NC	-
REVD	NC	-

* Compared with DEVD; NC, not cleaved (*k*_cat_/*K*_M_ <100 M^−1^ s^−1^).

Although the assay buffer contained reducing agents capable of disrupting disulfide bonds, the cysteine thiol may have been modified prior to the assay in ways that cannot be reversed by DTT. To evaluate this possibility, we used liquid chromatography-tandem mass spectrometry (LC-MS/MS) to assess potential post-translational modifications (PTMs) or oxidative states of the cysteine. The CEVD biosensor protein was denatured and reduced or not with DTT and alkylated or not with IAA to modify free cysteine residues, digested with trypsin/Lys-C, and analyzed by LC–MS/MS. We searched for the intact peptides GSAGTCEVDGK and GSAGTCEVDGKLPATMTSK, as well as cysteine-modified peptides, and used diagnostic fragment ions (underlined) to identify them. Unmodified intact peptides were detected in all samples tested (Supplementary Figure S4A,B and Supplementary Table S1). The presence of native peptides was further corroborated by the ability of IAA to alkylate the cysteine, forming carbamidomethyl-cysteine (Supplementary Figure S4C,D and Supplementary Table S1). Additional cysteine modifications observed included sulfenic (SOH), sulfinic (SO_2_H), and sulfonic (SO_3_H) acid forms (Supplementary Figure S4E–J and Supplementary Table S1); *S*-nitrosocysteine was not detected. From these results, we concluded that assays using the CEVD biosensor reflect a weighted average *k*_cat_/*K*_M_ value representing both modified and unmodified biosensor species (see the ‘Discussion’ section).

P_4_-Thr and -Ser biosensors follow P_4_-Cys biosensors in cleavage efficacy, with *k*_cat_/*K*_M_ values of 28,000 ± 800 M^−1^ s^−1^ and 26,000 ± 500 M^−1^ s^−1^, respectively. P_4_-His, -Glu, -Val, and -Asn form the next group in cleavage efficacy (*k*_cat_/*K*_M_ = 13,000–18,000 M^−1^ s^−1^), followed by a gradual decline in cleavage efficacy, with P_4_-Trp being the least efficaciously cleaved (*k*_cat_/*K*_M_ = 1700 ± 100 M^−1^ s^−1^). Biosensors containing a basic amino acid at P_4_ (Lys or Arg) were not cleaved by caspase-3 at enzyme concentrations up to 500 nM (k_cat_/*K*_M_ <100 M^−1^ s^−1^). Importantly, these two biosensors were readily cleaved by trypsin (Supplementary Figure S5), demonstrating that their cleavage sites were accessible to proteolysis.

We also compared cleavage of the P_4_ biosensor series by caspase-7, the closest homolog to caspase-3, and found that this peptidase is less promiscuous yet exhibits a similar overall cleavage profile (Supplementary Figure S6). Notable differences included the expected lower enzymatic activity of caspase-7 (∼4.3-fold lower than caspase-3), a shift in rank order whereby P_4_-Cys was cleaved less efficaciously than P_4_-Thr and -Ser, and the observation that only P_4_-Asp, -Ser, -Ala, -Thr, -Cys, -His, and -Glu variants were cleaved, whereas all others fell below the limit of detection (<100 M^−1^ s^−1^). Together, these findings further highlight the narrower specificity of the caspase-7 S_4_ pocket compared with that of caspase-3 (Supplementary Table S3).

Collectively, these results demonstrate that the BRET^2^ caspase biosensor assay is robust, provides reproducible quantification of proteolytic efficacy, and performs reliably across a wide range of cleavage efficacies, not only for optimal recognition sequences.

### Cellular use of caspase biosensors

**Figure 5:**
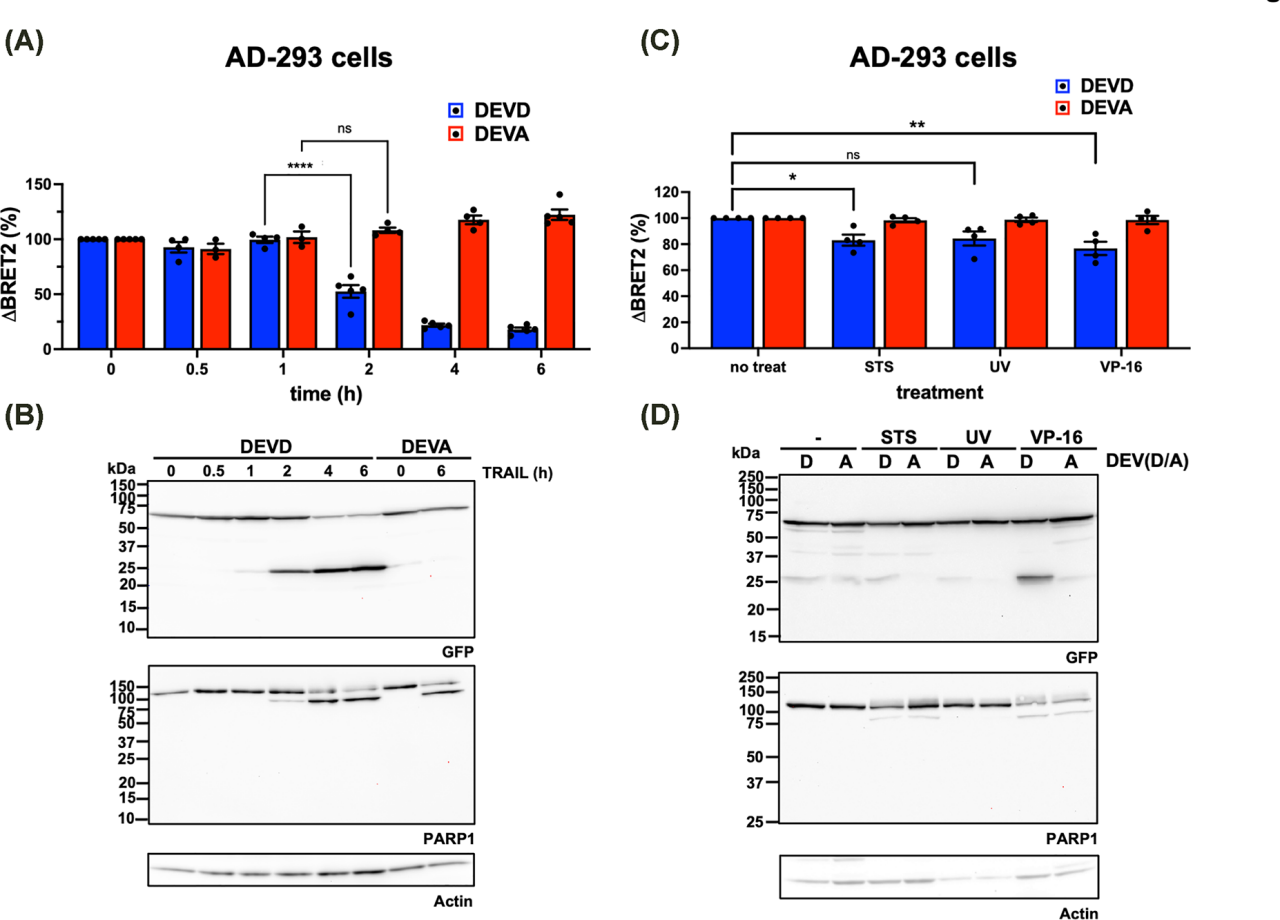
Cleavage of the DEVD biosensor in apoptotic AD-293 cells (**A**) Variation in BRET^2^ ratio in cells ectopically expressing DEVD (cleavable) and DEVA (uncleavable) biosensors. Cells were treated with 100 ng/ml TRAIL, and BRET^2^ ratio was measured after 6 h. *n* = 5. (**B**) Immunoblotting of samples similar as in panel (A) with anti-GFP and anti-PARP1 antibodies. Actin immunoblotting was used as a loading control. (**C**) Same as in panel (A) using STS (1 μM, 30 h), UV irradiation (100 J/m^−2^, harvested 30 h after irradiation), or VP-16 (100 μM, 30 h). *n* = 4. (**D**) Immunoblotting of samples similar as in panel (C) with anti-GFP and anti-PARP1 antibodies. Actin immunoblotting was used as a loading control. ns, not significant; *P-value <0.05; **P-value <0.01. Results are representative of at least two (B,D) independent experiments.

Because genetically encoded biosensors can be readily expressed in cells, we tested both DEVD and DEVA caspase biosensors in AD-293 cells exposed to the death ligand TRAIL (tumor necrosis factor α-related apoptosis-inducing ligand; Figure 5). In these cells, the BRET^2^ ratio decreased significantly by ∼50% at 2 h and fell to less than 25% at 6 h following TRAIL exposure (Figure 5A). The BRET^2^ ratio remained constant in cells expressing the DEVA biosensor, demonstrating that cleavage of the DEVD biosensor was specific to caspase activity and occurred at the expected P_1_–P_1′_ peptide bond.

Furthermore, the loss of BRET^2^ signal was mirrored by biosensor cleavage detected by immunoblotting with an anti-GFP antibody and closely tracked the cleavage of the caspase-3/7 substrate PARP1 (Figure 5B). STS and etoposide (VP-16), but not ultraviolet irradiation (UV), also caused a statistically significant decrease in the BRET^2^ ratio, although to a lesser extent than with TRAIL (Figure 5C). For these stimuli, the reduction in BRET^2^ ratio was similarly reflected by proteolysis of the biosensor and PARP1 (Figure 5D).

**Figure 6:**
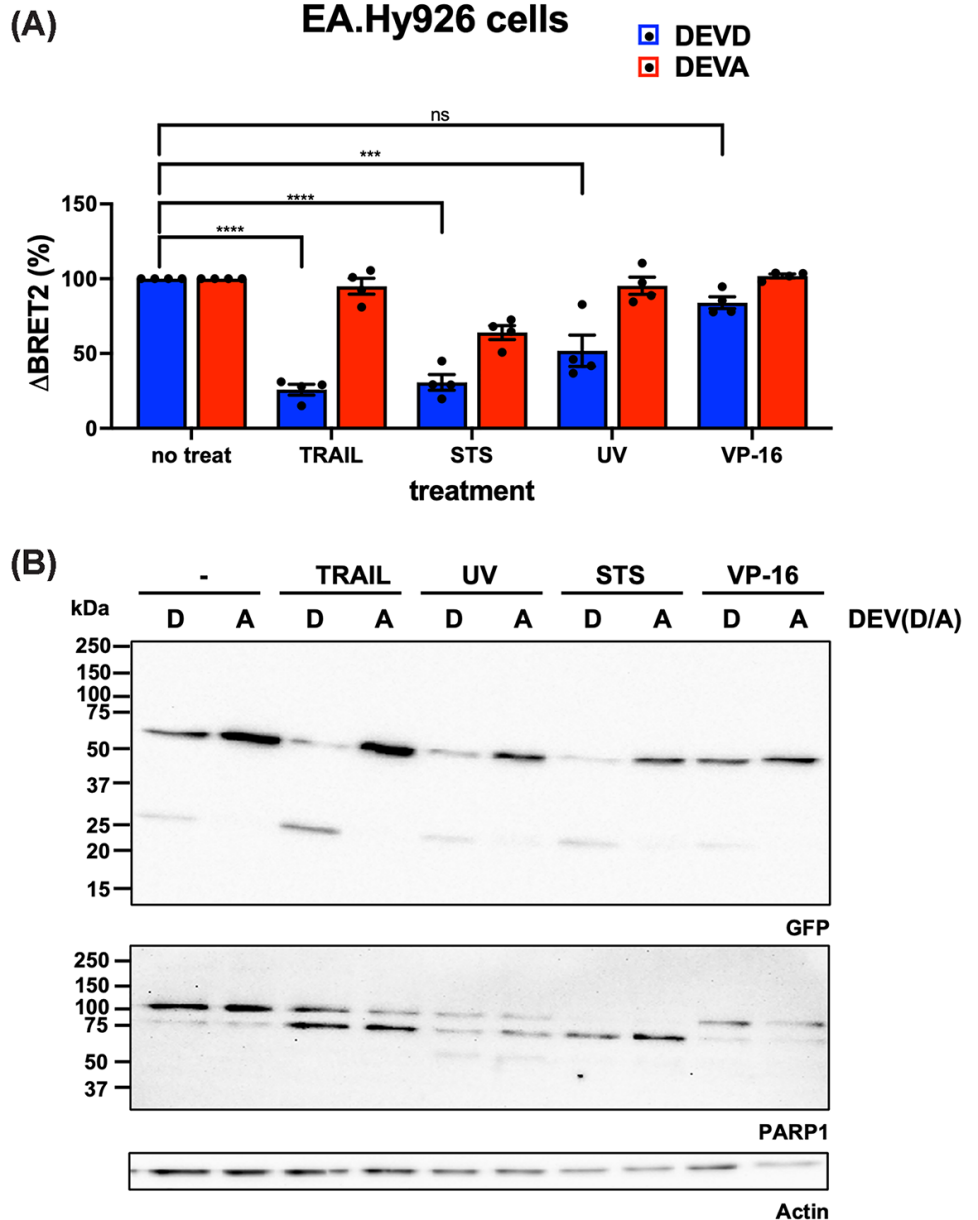
Cleavage of DEVD biosensor in apoptotic umbilical vein EA.hy926 cells (**A**) Variation in BRET^2^ ratio in EA.hy926 cells ectopically expressing DEVD (cleavable) or DEVA (uncleavable) biosensor. Cells were treated with TRAIL (100 ng ml^−1^, 6 h), UV irradiation (100 J m^−2^, harvested 24 h after irradiation), STS (1 μM, 24 h), or VP-16 (100 μM, 24 h). *n* = 4. (**B**) Immunoblotting of samples similar as in A) with anti-GFP and anti-PARP1 antibodies. Actin immunoblotting was used as a loading control. ns, not significant; ***P-value <0.001; ****P-value <0.0001. Results are representative of three (B) independent experiments.

We further demonstrated that TRAIL, STS, and UV—but not VP-16—caused a statistically significant loss of BRET^2^ signal in the somatic hybrid endothelial EA.hy926 cell line (Figure 6). These cells were particularly sensitive to STS, resulting in loss of maximal BRET^2^ ratio, as shown by signal reduction in cells expressing the DEVA biosensor. Despite the intrinsic limitations of turn-off biosensors (see the ‘Discussion’ section), the BRET^2^ caspase biosensors provided a reliable measure of relative caspase activity during apoptosis.

## Discussion

We employed a simple turn-off BRET^2^-based caspase biosensor to investigate cleavage site motifs in a proteinaceous context. Similar in principle to the nanoLuc/mNeonGreen system [68], our biosensor combines *Renilla* luciferase and a modified GFP from *Aequorea victoria*, neither of which are natural caspase substrates. This ensures an unbiased means for testing peptide motifs. While the BRET^2^ biosensor is more complex to use than small peptide substrates, it provides a protein-based readout that better reflects genuine caspase targets.

The BRET^2^ biosensor can be used as purified protein *in vitro*, in cell lysates, and in living cells, and reporters using bioluminescence have been applied to study various biological processes, including signaling pathways, in both light-on and light-off configurations (reviewed in [77]). The main advantage of ours lies in its simplicity: the linker containing the caspase cleavage site serves only to maintain proximity between GFP10 and *R*LucII. In contrast, other caspase biosensors, such as iCasper [63] and split-luciferase systems [65], incorporate cleavage sites within segments that impose structural constraints (a potential source of bias) to physically separate fluorescent and luciferase proteins. Moreover, the split-luciferase biosensor requires cleavage at two sites to generate a signal [65], making kinetic studies unpractical even when both cleavage sites are identical. Similar limitations apply to biosensors using circular permutation of fluorescent proteins, like Venus [78]. By comparison, the straightforward GFP10–*R*LucII BRET^2^ design reduces the risk of structural bias. Nevertheless, some bias may still occur, which could be assessed by reproducing the kinetic experiments with another, unrelated, genetically encoded biosensor system.

Overall, the results from the P_4_ biosensor series align with the P_4_ preferences observed in studies using peptidic substrates (Supplementary Table S2), supporting the assumption that the BRET^2^ biosensors provide an unbiased assessment.

As expected, P_4_-Asp is preferred, as shown by both peptide-based assays and proteomic analyses. Previous studies using fluorogenic and internally quenched peptide substrates demonstrated that caspase-3 cleaves sequences with P_4_-Asp at rates at least 10 times higher than those with P_4_-Glu, -Ser, or -Thr [41,79] and exhibits nearly 100-fold greater catalytic specificity [42]. Proteomic studies further confirm this preference, whether focusing on caspase-3 specifically [47] or surveying the entire caspase degradome in apoptotic cells [80–82].

P_4_-Cys ranked second in cleavage efficacy, which was remarkable given that this residue is rarely represented in peptide substrate libraries [83]. The intermediate cleavage efficacy of the CEVD biosensor—between DEVD and TEVD/SEVD—may reflect cysteine PTMs, particularly oxidation to sulfinic acid, which makes it closely resemble aspartate. We detected cysteine oxidation products, but the diversity and relative abundance of modified species could not be resolved, as some forms require targeted stabilization or enrichment [84]. Under physiological conditions, the reducing cytosol limits cysteine modification, but during apoptosis the environment becomes less reducing [85,86], permitting dynamic PTMs such as S-nitrosylation [87], which appears incompatible with efficacious caspase cleavage [88]. In contrast, oxidation to sulfinic acid—reversible by sulfiredoxin—could enhance caspase-3 proteolysis by rendering P_4_-Cys ‘aspartate-like’. Consistent with this possibility, nearly 50 caspase substrates containing a P_4_-Cys are listed in CaspSites substrate database [89]. Together, these observations suggest that PTMs at P_4_ may constitute a regulatory layer in caspase-mediated proteolysis. The contribution of specific cysteine PTM to proteolysis efficacy may be best assessed using peptide-based substrates. which can incorporate unnatural residues.

After P_4_-Cys, -Thr, and -Ser, caspase-3 exhibits varying cleavage efficacies for other residues, except Arg and Lys, with P_4_-Trp showing the lowest cleavability. Our data also indicate that caspase-3 cleaves motifs with a P_4_ residue preferred by other caspases, including Val (caspase-6), Ile (caspases-8 and -10), Leu (caspases-9 and -10) [39–42]; it even retains activity on motifs favored by inflammatory caspases (P_4_-Tyr) and caspase-14 (P_4_-Trp). In contrast, caspase-7’s activity was only measured for P_4_-Asp, -Ser, -Thr, -Ala, -Cys, -His, and -Glu, further highlighting its narrower specificity compared with caspase-3. Unless this order is due to an error in measurement (overall large error bars), the fact that P_4_-Cys is cleaved less efficaciously than P_4_-Ser, -Thr, and -Ala indicates that caspase-7 may not accept modified cysteine residues in its S_4_ pocket as well as caspase-3. These findings support our hypothesis that caspase-3 acts as a ‘generalist’ executioner caspase, whereas caspases-6 and -7 are specialized enzymes with a restricted degradome [35].

Fewer than 20 proteins listed in CaspSites [89] are cleaved at sites containing a P_4_-Arg or -Lys, including the human ataxin-2-like protein (ATX2L), which is cleaved at KEVD_584_↓S in various cell lines undergoing apoptosis. These examples underscore the need to study the structural context of caspase proteolysis and the possibility that very unfavorable sites are cleaved with assistance from exosites [49]. The promiscuity of the caspase-3 S_4_ pocket, which accommodates nearly all residues, may help regulate the timing of proteolysis during apoptosis.

Notably, the differences in cleavage efficacy observed with protein-based biosensors were much narrower than those reported for short peptides. For example, the difference between P_4_-Asp and -Thr was only 2.1-fold, compared with more than 70-fold for internally quenched Abz/3-nitroTyr peptides; similarly, P_4_-Pro showed a 14-fold difference *versus* 1000-fold in peptide assays [42]. The quenched peptides from the that study have aminobenzoic acid (Abz)-Gly in P_6_–P_5_, whereas other studies have peptides bearing an acetylated N-terminus that removes the positive charge on the N-terminus, mimicking natural proteins. It is not clear how these non-natural extensions may affect proteolysis efficacy within each series of peptides, but it remains that motif presentation may be different than within protein biosensors. If so, it reinforces the idea that structural context—absent in short peptides—influences cleavage efficacy beyond the canonical substrate-binding site and may favor or disfavor specific residues.

Interestingly, our previous work also showed much less discrimination between P_4_-Asp and other residues in engineered p23 proteins for caspase-7 proteolysis [90]. Although it is difficult to disentangle the contribution of the chaperone activity of p23 with residue-specific effects, caspase-7 cleaved a modified p23 protein bearing an optimal DEVD_142_↓G site 7-fold more efficaciously than the native p23 PEVD↓G site. In contrast, Stennicke and colleagues reported a 16,500-fold difference using internally quenched fluorogenic peptides [42].

Poreba and colleagues compiled data on the cleavage of the same DEVD tetrapeptide by caspase-3 with different leaving groups and found at least a 10-fold range in cleavage efficacy [91]. This variability suggests that regions outside the canonical DEVD recognition motif, especially residues on the prime side of the scissile bond, can substantially influence caspase activity. Evidence for this remains limited, but notable examples include the contribution of a P_5_-Val residue to improved catalysis by caspase-2 [36] and the influence of P_6_, P_5_, P_2′_, and P_3′_ residues on caspase-7 specificity [81]. In the latter study, the authors proposed that caspase-7 achieves specificity not by stronger substrate binding but by avoiding substrates recognized by caspase-3, supporting the concept of an extended substrate-binding pocket. Protein-based biosensors offer a practical approach to assess positions beyond the canonical P_4_–P_1′_ region.

Finally, the BRET^2^ biosensor is suitable for use in intact cells, but its use is more limited because they cannot report kinetics like they do *in vitro*. As apoptosis progresses asynchronously over many hours within a cell population, and caspase-3 activation varies depending on factors such as cell cycle and lineage [92,93], cell type [94,95], and the apoptotic stimulus [95–97], during the 30-min BRET^2^ assay, measurements reflect the average caspase activity across all cells rather than *k*_cat_/*K*_M_ values for caspase-3. Because the biosensor contains the preferred cleavage motif of executioner caspases-3 and -7, the assay primarily reports their combined activity but also that of other caspases to some extent. Nevertheless, the BRET^2^ biosensor reliably reflects the extent of apoptosis, as confirmed by PARP1 proteolysis.

## Conclusion

We demonstrate that the BRET^2^ biosensor provides a robust, highly adaptable, and unbiased platform for assessing caspase substrate preferences in a protein context while delivering reproducible kinetic data. Using it, we further showed that the range of cleavage efficacies in protein substrates is markedly narrower than that previously observed with peptide-based assays, for reasons that remain unclear. This observation warrants further investigation using alternative unbiased biosensors and engineered protein substrates. For example, Timmer and colleagues showed that optimizing a loop in carA (the small chain of *E. coli* carbamoyl phosphate synthase) containing an accidental caspase cleavage site resulted in a 200-fold increase in cleavage efficacy [76], suggesting that similar strategies could be used to reproduce the P_4_ library and expand our findings using protein-based biosensors.

## Supplementary Material

Supplementary Figures S1-S6 and Tables S1-S3

## Data Availability

Datasets are available on request. The raw data supporting the conclusions of this article will be made available by the authors, without undue reservation.

## Abbreviations

ACC: 7-amino-4-carbamoylmethylcoumarin
DTT: dithiothreitol
BRET: Bioluminescence resonance energy transfer
BRET^2^: double brilliance bioluminescence resonance energy transfer
FRET: Förster resonance energy transfer
IMAC: immobilized metal affinity chromatography
IAA: iodoacetamide
LC-MS/MS: liquid chromatography-tandem mass spectrometry
MRM: multiple reaction monitoring
PARP1: poly(ADP-ribose) polymerase 1
STS: staurosporine
TRAIL: tumor necrosis factor α-related apoptosis-inducing ligand
Z-VAD-fmk: Benzyloxycarbonyl-VAD-fluoromethyl ketone
