# Supplementary material for: Using a bioluminescence resonance energy transfer caspase biosensor to study caspase-3 cleavage site specificity
Source: Biosci Rep. 2026 Mar 24;46(4):BSR20254030. doi: 10.1042/BSR20254030 (PMC13071377; doi:10.1042/BSR20254030)
Supplement: Supplementary Figures S1-S6 and Tables S1-S3 [file BSR-2025-4030_supp.pdf]

A

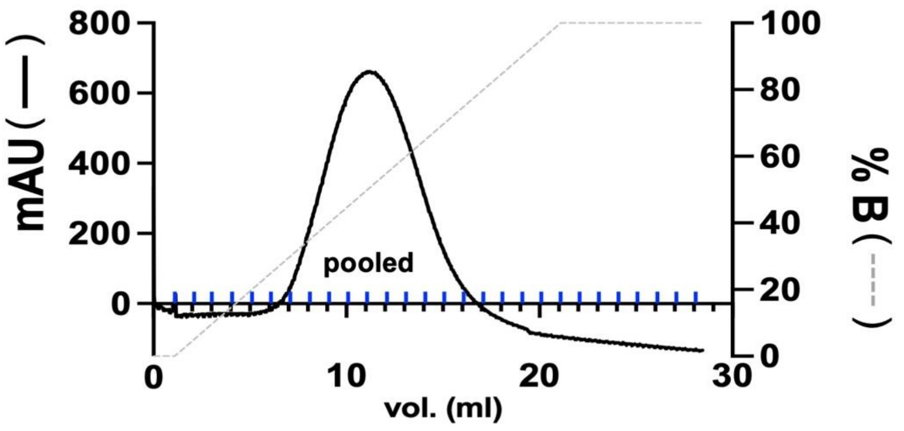

B

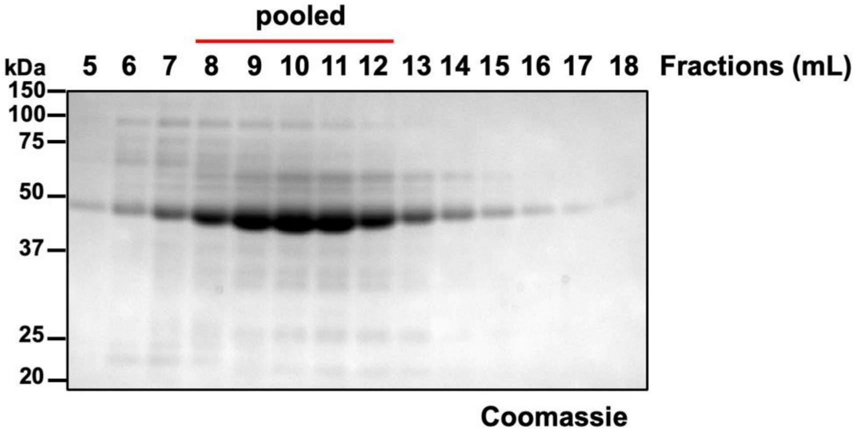

Supplementary Figure 1. Purification of the GFP10-DEVD↓G-*R*LucII (DEVD biosensor). **A)** Representative elution profile of the DEVD biosensor from the IMAC column. One hundred % B is 200 mM imidazole (gray dash line). **B)** Ten µL of fractions from A) were analyzed by SDS-PAGE. Red line indicates pooled fractions. All biosensors were purified similarly. All P_4 biosensors were produced with similar purification result.

A

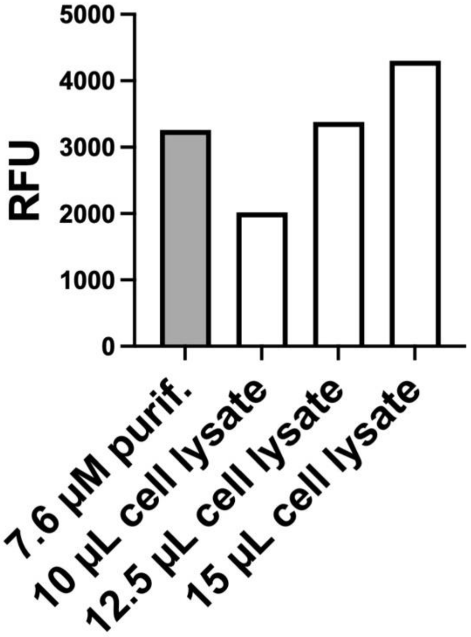

B

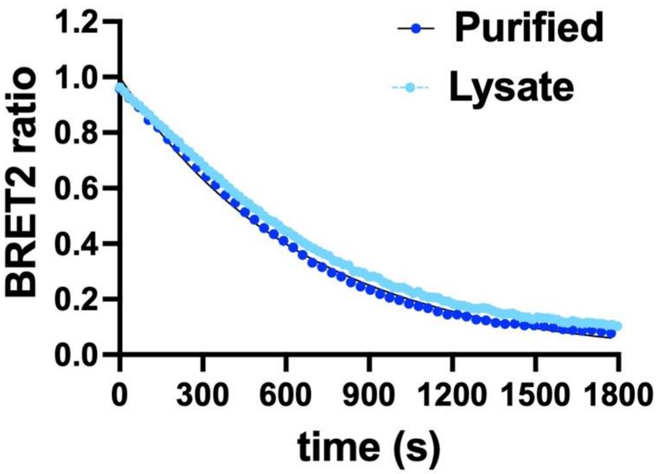

DEVD biosensor	Calculated k ($M^{-1}s^{-1}$)
Purified	60,267
From lysate	53,950

Supplementary Figure 2. The DEVD biosensor in lysate. **A)** A reference preparation of GFP10 was used to titrate the concentration of biosensor in lysate (EX_λ = 395, EM_λ = 510). **B)** Cleavage by 25 nM caspase-3 of 10 nM purified DEVD biosensor compared to an amount of lysate established using data from A). *k* values were calculated as described in **Materials and methods**. Results are representative of two independent experiments.

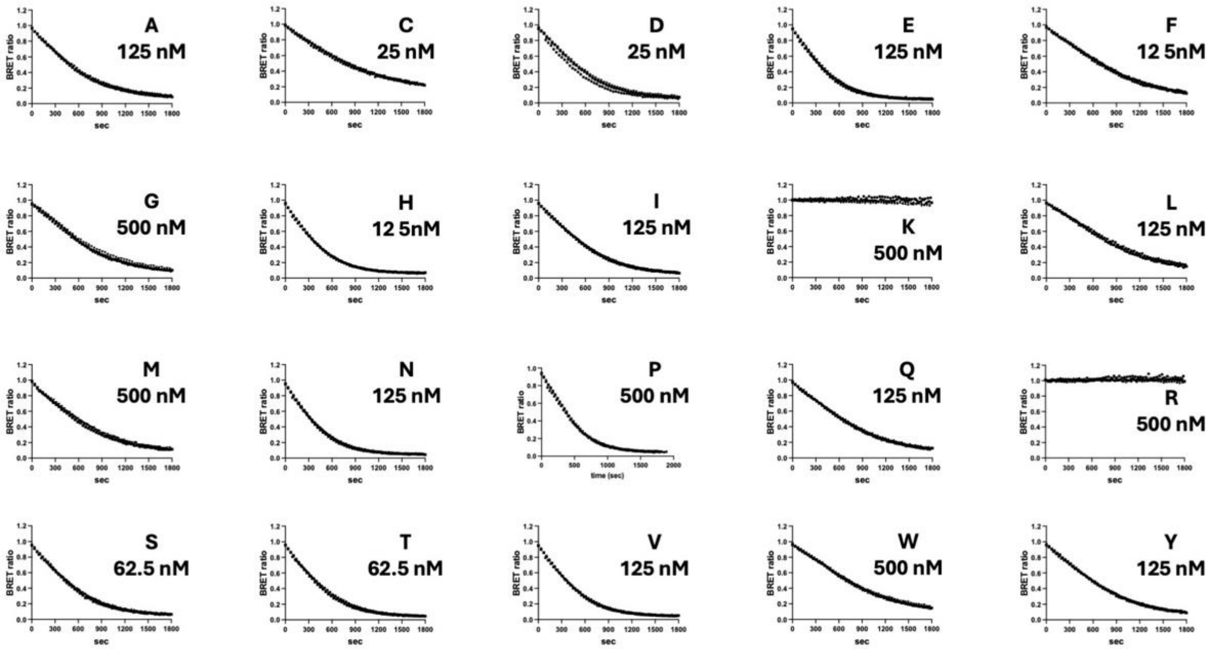

Supplementary Figure 3. Representative dataset of P_4 biosensor library cleavage by the indicated concentration of caspase-3. Results from replicates were reported in **Fig. 4C** and **Tab. 1**.

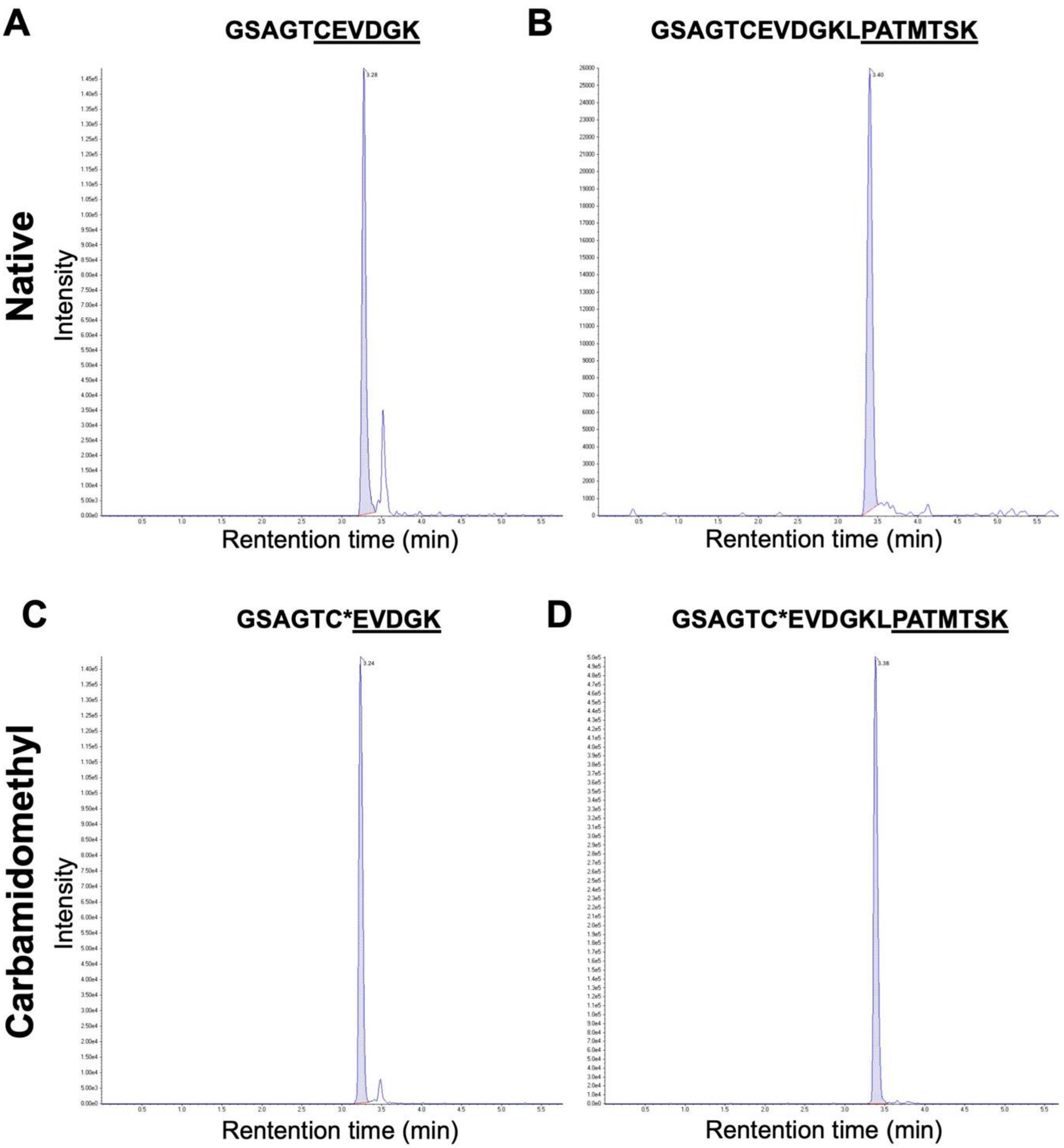
A
GSAGTCEVDGK
B
GSAGTCEVDGKLPATMTSK
Native
Intensity
3.28
3.40
Rentention time (min)
Rentention time (min)
C
GSAGTC*EVDGK
D
GSAGTC*EVDGKLPATMTSK
Carbamidomethyl
Intensity
3.24
3.38
Rentention time (min)
Rentention time (min)

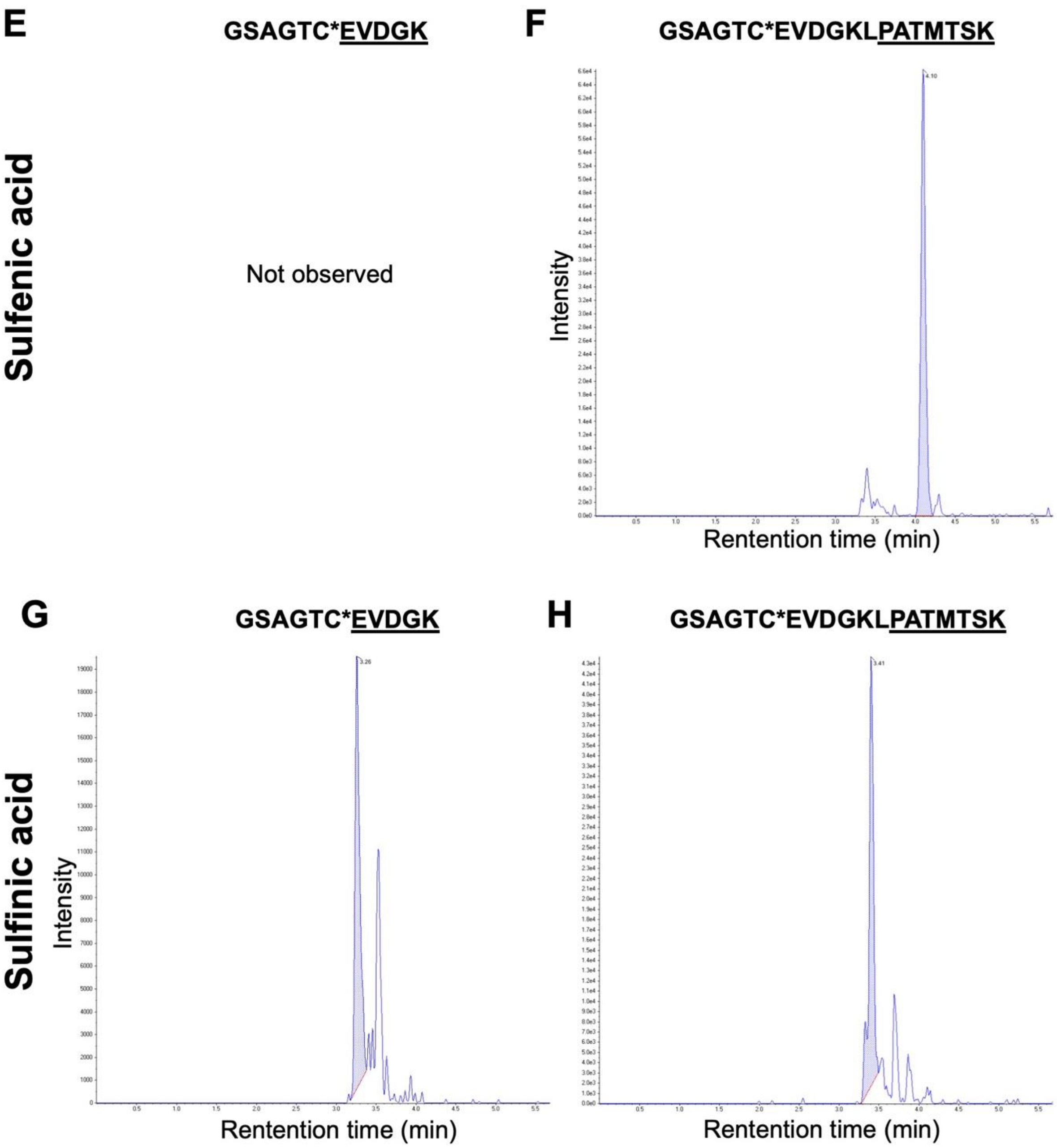
E
GSAGTC*EVDGK
F
GSAGTC*EVDGKLPATMTSK
Sulfenic acid
Not observed
Intensity
4.10
Rentention time (min)
G
GSAGTC*EVDGK
H
GSAGTC*EVDGKLPATMTSK
Sulfinic acid
Intensity
3.26
3.41
Rentention time (min)
Rentention time (min)

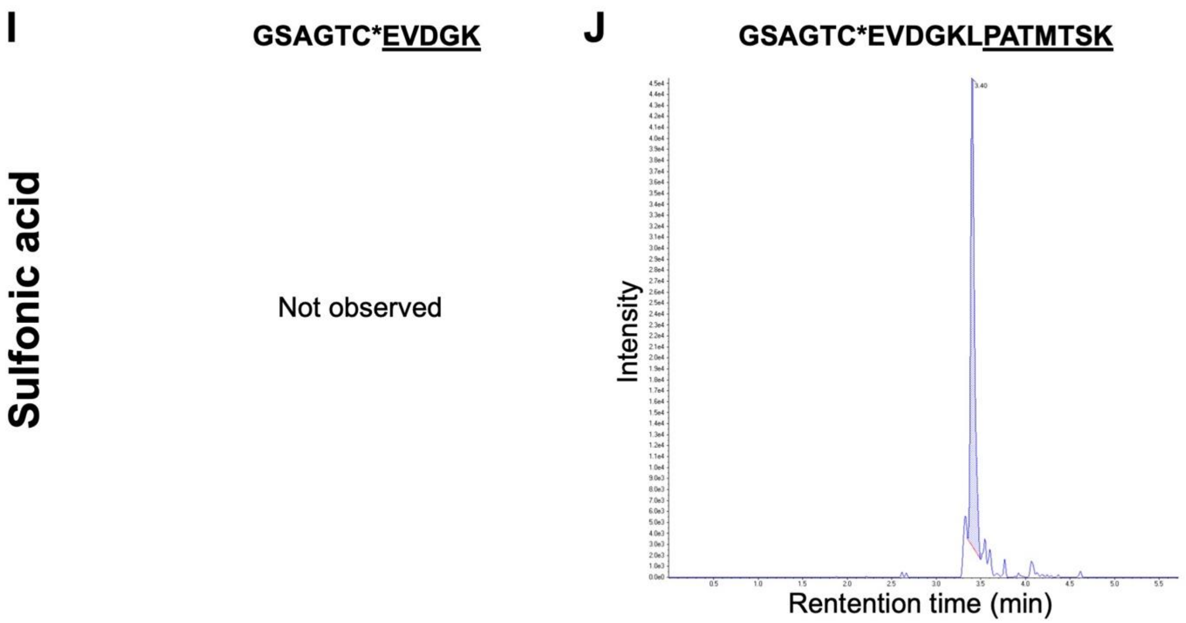

Supplementary Figure 4. Modified-cysteine residue in the CEVD biosensor. CEVD BRET^2 biosensor was denatured with urea and digested with Trypsin/Lys-C. Fragments from the modified GSAGTC*EVDGK (**A**, **C**, **E**, **G**, **I**) and GSAGTC*EVDGKLPATMTSK (**B**, **D**, **F**, **H**, **J**) peptides were searched by mass spectrometry with intact or modified cysteine (*). Chromatograms for native peptides (**A**, **B**), IAA-alkylated native peptides (**C**, **D**), sulfenic (SOH; **E**, **F**), sulfinic (SO_2H; **G**, **H**), and sulfonic (SO_3H; **I**, **J**) acid-modified cysteine-containing peptides. A reference peptide fragment (underlined) from the various modified peptides was used for identification (**Tab. S1**). Samples were analyzed three times; not all modifications were observed in each analysis.

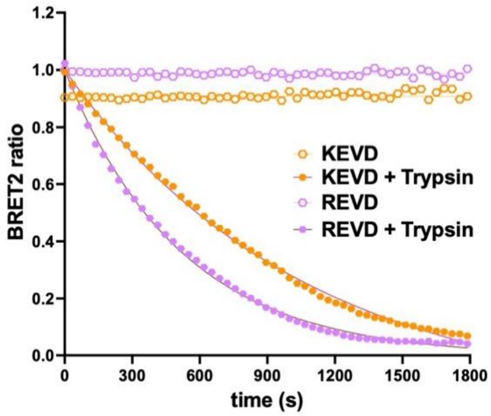

Supplementary Figure 5. Biosensors containing a P_4-Lys/Arg residue are cleavable. Cleavage by 500 nM trypsin of 10 nM purified KEVD or REVD biosensor was analyzed for $BRET^2$ ratio signal decrease. Results are representative of two independent experiments.

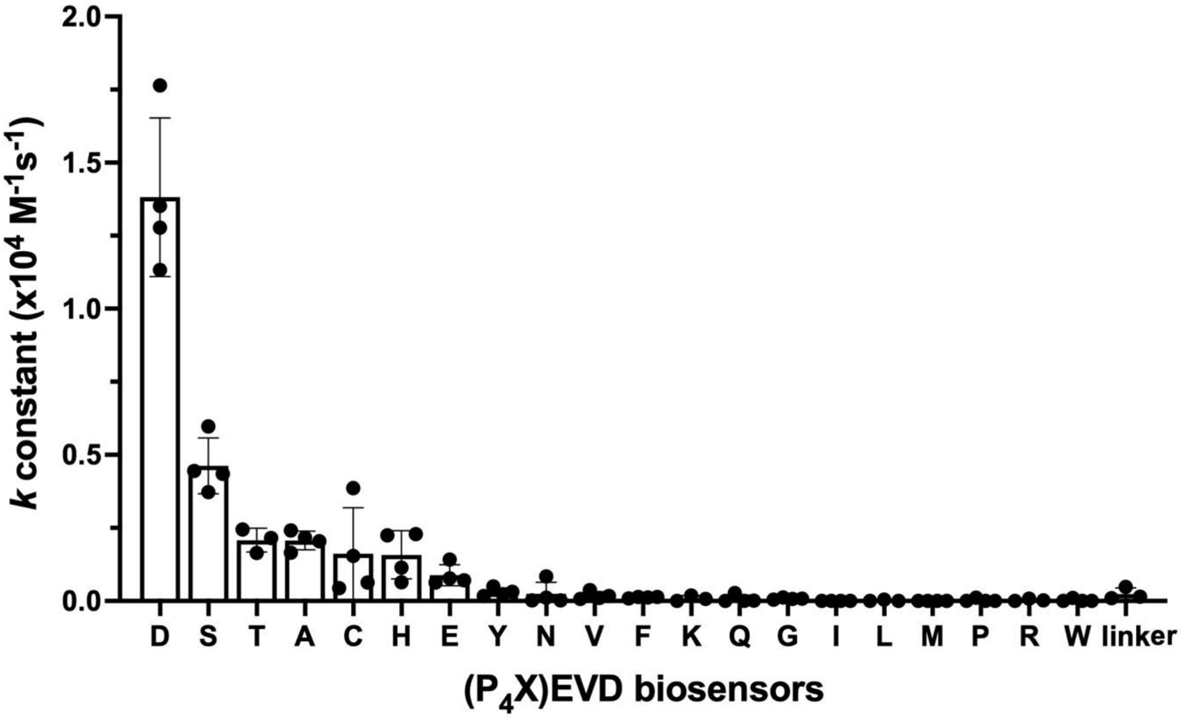

Supplementary Figure 6. P_4 caspase biosensor library cleaved by caspase-7. Calculated *k* values for the cleavage by caspase-7 of each (P_4X)EVD biosensor using assays as described in **Fig. 3B**. N = 4.

Supplementary Table 1. Chromatographic data for peptides.

Modifications	Peptides[1]	Ions Da[2]	Retention time (min)[3]
Native	GSAGTCEVDGK	650.3 (y6)	3.28
Carbamidomethyl	GSAGTC(CAM)EVDGK	547.3 (y5)	3.24
S-Nitroso	GSAGTC(SNO)EVDGK	547.3 (y5)	n.i.
Sulfenic acid	GSAGTC(SOH)EVDGK	547.3 (y5)	n.i.
Sulfinic acid	GSAGTC(SO_2H)EVDGK	547.3 (y5)	3.26
Sulfonic acid	GSAGTC(SO_3H)EVDGK	547.3 (y5)	n.i.
Native	GSAGTCEVDGKLPATMTSK	735.4 (y2)	3.40
Carbamidomethyl	GSAGTC(CAM)EVDGKLPATMTSK	735.4 (y2)	3.38
S-Nitroso	GSAGTC(SNO)EVDGKLPATMTSK	735.4 (y2)	n.i.
Sulfenic acid	GSAGTC(SOH)EVDGKLPATMTSK	735.4 (y2)	4.10
Sulfinic acid	GSAGTC(SO_2H)EVDGKLPATMTSK	735.4 (y2)	3.41
Sulfonic acid	GSAGTC(SO_3H)EVDGKLPATMTSK	735.4 (y2)	3.40

n.i. : This peptide was not identified.

[1] The underlined peptides were used for identification.

[2] Some fragment ions (notably y5 and y2) are shared between several peptide forms (modified or not), which limits their specificity. This makes interpretation more complex when relying solely on a shared fragment signal.

[3] Overlapping retention times between certain modifications, or incomplete cleavage, can mask certain forms or falsely suggest their presence.

Supplementary Table 2. Comparison of the P_4 preference between studies for caspase-3.

**This study**	**Poreba *et al.* (2017)**[1]	**Stennicke *et al.* (2000)**[2]	**Thornberry *et al.* (1997)**[3]
BRET[2] biosensors	Fluorogenic peptidic substrates	Internally-quenched peptidic substrates	Fluorogenic peptidic substrates
**Asp**	**Asp**	**Asp**	**Asp**
Cys*			
**Thr**	**Thr**	**Thr**	**Thr**
**Ser**	**Ser**	**Ser**	**Ser**
His			
**Glu**	**Glu**	**Glu**	**Glu**
**Val**	Met	Tyr	**Val**
**Asn**	**Asn**	Val	**Asn**
Ala	His	Phe	
Ile	Val	Asn	
**Tyr**	**Tyr**	Gln	
Gln	Ala	Ala	
Phe	Ile	Leu	
**Leu**	**Leu**	Gly	
**Pro**	**Pro**	**Pro**	
Met		-	
**Gly**	**Gly**	-	
Trp		-	
**Arg**	**Arg**	**Arg**	
**Lys**	**Lys**	**Lys**	
	Trp		

[1]: In the absence of numerical data, order was based on **Fig. 4** from (1).
[2]: In the absence of numerical data, order was based on **Fig. 1** from (2).

1. Poreba M, Salvesen GS, Drag M. Synthesis of a HyCoSuL peptide substrate library to dissect protease substrate specificity. Nat Protoc. 2017;12(10):2189-214. 10.1038/nprot.2017.091
2. Stennicke HR, Renatus M, Meldal M, Salvesen GS. Internally quenched fluorescent peptide substrates disclose the subsite preferences of human caspases 1, 3, 6, 7 and 8. Biochem J. 2000;350 Pt 2(Pt 2):563-8. 10.1042/bj3500563
3. Thornberry NA, Rano TA, Peterson EP, Rasper DM, Timkey T, Garcia-Calvo M, et al. A combinatorial approach defines specificities of members of the caspase family and granzyme B. Functional relationships established for key mediators of apoptosis. J Biol Chem. 1997;272(29):17907-11. 10.1074/jbc.272.29.17907

Supplementary Table 3. Cleavage efficacy of the P_4 series of $BRET^2$ biosensors by caspase-7.

Motifs	K_{cat}/K_M ($M^{-1} \cdot s^{-1}$) ± SEM	Fold less than DEVD[1]
DEVD	14,000 ± 1,400	1.0
SEVD	4,600 ± 500	3.0
TEVD	2,100 ± 200	6.7
AEVD	2,100 ± 200	6.7
CEVD	1,600 ± 800	8.8
HEVD	1,600 ± 400	8.8
EEVD	880 ± 200	15.9
FEVD	NC	-
GEVD	NC	-
IEVD	NC	-
KEVD	NC	-
LEVD	NC	-
MEVD	NC	-
NEVD	NC	-
PEVD	NC	-
QEVD	NC	-
REVD	NC	-
VEVD	NC	-
WEVD	NC	-
YEVD	NC	-

[1]: compared to DEVD; NC, not cleaved (k_{cat}/K_M <100 $M^{-1} \cdot s^{-1}$).
